# Age-dependent remodelling of arterial chemoafferent innervation in hypertension

**DOI:** 10.1093/cvr/cvaf207

**Published:** 2025-10-29

**Authors:** Audrys G Pauza, Igor Felippe, Iris de Laat, Olivia Gold, Xin Shen, Julian F R Paton

**Affiliations:** Manaaki Mānawa—The Centre for Heart Research, Department of Physiology, Faculty of Medical & Health Sciences, University of Auckland, 85 Park Road, Grafton, Auckland 1023, New Zealand; Manaaki Mānawa—The Centre for Heart Research, Department of Physiology, Faculty of Medical & Health Sciences, University of Auckland, 85 Park Road, Grafton, Auckland 1023, New Zealand; Manaaki Mānawa—The Centre for Heart Research, Department of Physiology, Faculty of Medical & Health Sciences, University of Auckland, 85 Park Road, Grafton, Auckland 1023, New Zealand; Manaaki Mānawa—The Centre for Heart Research, Department of Physiology, Faculty of Medical & Health Sciences, University of Auckland, 85 Park Road, Grafton, Auckland 1023, New Zealand; Manaaki Mānawa—The Centre for Heart Research, Department of Physiology, Faculty of Medical & Health Sciences, University of Auckland, 85 Park Road, Grafton, Auckland 1023, New Zealand; Manaaki Mānawa—The Centre for Heart Research, Department of Physiology, Faculty of Medical & Health Sciences, University of Auckland, 85 Park Road, Grafton, Auckland 1023, New Zealand

**Keywords:** Hypertension, Innervation, Carotid body, Arterial chemoreflex, Sympathetic activity

## Abstract

**Aims:**

Elevated sympathetic nerve activity (eSNA) is a hallmark of cardiovascular disease and represents an important clinical target for disease management. A known driver of eSNA is aberrant signalling from, and sensitization of, the carotid body (CB) arterial chemoreceptors. Sensitization is coupled with CB hypertrophy, the cause of which remains unclear. Here, we set out to characterize the morphological basis of CB hypertrophy in hypertension to understand its aberrant activity.

**Methods and results:**

Using high-throughput fluorescence microscopy, we mapped the neurovascular interface and chemoafferent innervation of the CB of spontaneously hypertensive rats (SHR) across multiple age groups. We show that CB hypertrophy driven by chemosensory (type I) cell hyperplasia and an expanded vascular network is evident in 4–6-week-old SHR without established hypertension. Specifically, CB hypertrophy in the SHR is linked to increased chemoafferent innervation and an age-dependent remodelling of nerve fibre composition.

**Conclusions:**

CB hypertrophy in hypertension is associated with chemosensory hyperplasia and angiogenesis, likely mediated by impaired HIF-PHD signalling in the SHR. We propose that CB size may serve as a candidate marker of chemoafferent sensitivity and the efficacy of therapies targeting the CB. However, further validation in humans is needed to support this link. Neurotrophic pathways promoting increased chemoafferent innervation in hypertension are proposed as a potential target for modulating CB activity in sympathetically mediated diseases.

## Graphical Abstract

**Graphical Abstract:**
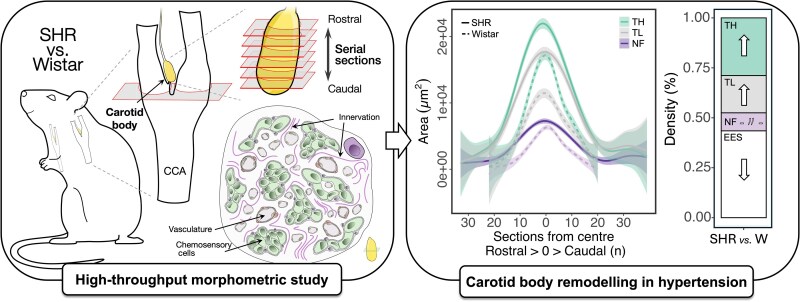

**Time of primary review: 52 days**

**See the editorial comment for this article ‘Hypertensive remodelling: when bigger body means bigger problems’, by E. Lazartigues, https://doi.org/10.1093/cvr/cvaf245.**

## 1. Introduction

Hypertrophy of the carotid body (CB) arterial chemoreceptors is an established clinical feature of essential hypertension, type 2 diabetes mellitus, and chronic heart failure.^1–4^ CB hypertrophy is also observed in preclinical models of neurogenic hypertension,^5^ high-caloric diet induced insulin resistance,^6^ and chronic hypoxia.^7^ Increased CB size is coupled with peripheral chemoafferent sensitization that drives excess sympathetic nerve activity (eSNA).^8^ This represents an important clinical target for managing cardiometabolic disease as uncontrolled eSNA exacerbates cardiovascular risk and deteriorates prognosis.^9^ Interventions targeting the CB, either surgical removal or transcatheter CB denervation, have been effective at reducing eSNA and lowering blood pressure in treatment-resistant, grade 3 hypertension.^10,11^ Notwithstanding the therapeutic benefit, the aetiological mechanisms behind CB sensitization in cardiac disease, coupled with the organ’s hypertrophy, remain elusive.

The spontaneously hypertensive rat (SHR) is a model of peripheral chemoafferent sensitization, and several studies have examined anatomical changes linked to CB hypertrophy.^12–18^ However, no clear consensus has emerged. While some attribute CB hypertrophy to chemosensory (type I) cell proliferation (hyperplasia) without changes in area-normalized cell density,^14,15^ others report a reduction in chemosensory cell count or no change.^12,16^ Similarly, evidence remains inconclusive regarding alterations to the vasculature in the CB of SHR.^12,16,17^ To date, no study has examined changes in all chemosensory components simultaneously while accounting for the organ’s three-dimensional structure. This forms the aim of this study.

**Figure 1:**
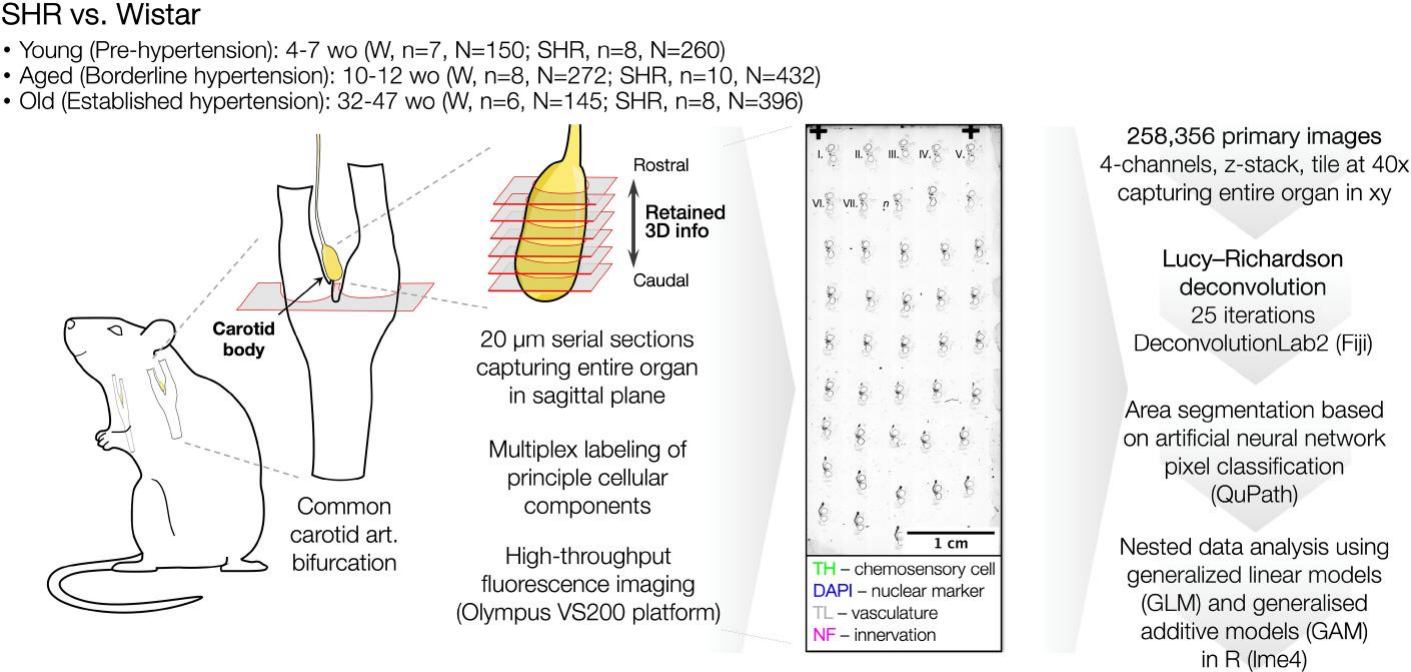
Study overview. Carotid bodies from SHR and Wistar controls were analysed across different age groups. The common carotid artery bifurcation (unilateral) was serially sectioned at 20 µm in the sagittal plane, with sections mounted in sequence (roman numerals on a representative slide) to permit reconstruction of rostral–caudal directionality following image acquisition. Multiplex fluorescent labelling targeted key components: chemosensory (glomus) cells (TH—tyrosine hydroxylase), vasculature (TL—tomato lectin), and innervation (NF—160 kDa neurofilament). Imaging was performed using a high-throughput widefield fluorescence microscopy platform (Olympus VS200). Lucy–Richardson deconvolution was applied, and binary area segmentation was performed using an artificial neural network-based pixel classification in QuPath. Hypothesis testing was performed using generalized additive models (GAMMs) and generalized linear models (GLMs) to account for a multi-group, nested data structure.

Our group recently showed that the peripheral chemoafferent sensitization involves aberrant sympathetic drive to the CB itself, resulting in a feed-forward amplification of chemoafferent sensitivity in the SHR.^19^ This builds on our earlier finding that increased CB sensitivity to hypoxia in the SHR is linked to elevated P2X3 receptor levels on petrosal sensory terminals innervating the organ.^20,21^ However, it remains unclear whether this relates to an increased arborization of the chemosensory/sympathomotor innervation (a change in the number of fibres) or altered synthesis of the purinergic receptors with the innervation density remaining constant. Only two prior studies have quantified CB innervation in the SHR, reporting a net reduction in neuropeptide-containing varicosities corresponding to both sensory (CGRP, SP) and motor (NPY) nerve fibres.^17,22^ Such a reduction appears inconsistent with our functional data, which indicate an overall increase in sympathomotor drive to the CB,^19^ highlighting the need for further investigation. To this end, we performed a high-throughput morphometric analysis of principal CB structural components and innervation in the SHR compared with age-matched normotensive Wistar rats (*Figure 1*).

## 2. Methods

### 2.1 Animals

A total of 52 age-matched, male Wistar (*n* = 22) and SHR (*n* = 26) rats were used in the study (*Table 1*). Animals were purchased from the Vernon Jansen Unit (University of Auckland, New Zealand) and housed in conventional Tecniplast 1500 rat cages with controlled temperature (21 ± 2°C), humidity (55 ± 10%), and 12 h light–dark cycle. Animals had unlimited access to food and water. All studies were approved by the University of Auckland committee for the ethical use of animals in scientific research (AEC# 2274) and conducted in accordance with Directive 2010/63/EU of the European Parliament on the protection of animals used for scientific purposes.

**Table 1:** Animals used in the study

Strain	Group	ID	Age (days)	Strain	Group	ID	Age (days)
SHR	Young	SY1	45.0	Wistar	Young	WY1	36.0
SHR	Young	SY2	45.0	Wistar	Young	WY2	36.0
SHR	Young	SY3	45.0	Wistar	Young	WY3	36.0
SHR	Young	SY4	45.0	Wistar	Young	WY4	36.0
SHR	Young	SY5	45.0	Wistar	Young	WY5	37.0
SHR	Young	SY6	48.0	Wistar	Young	WY6	37.0
SHR	Young	SY7	48.0	Wistar	Young	WY7	37.0
SHR	Young	SY8	48.0	Wistar	Young	WY8	27.0
SHR	Aged	SA1	74.0	Wistar	Aged	WA1	74.0
SHR	Aged	SA2	74.0	Wistar	Aged	WA2	74.0
SHR	Aged	SA3	77.0	Wistar	Aged	WA3	74.0
SHR	Aged	SA4	73.0	Wistar	Aged	WA4	74.0
SHR	Aged	SA5	73.0	Wistar	Aged	WA5	76.0
SHR	Aged	SA6	73.0	Wistar	Aged	WA6	74.0
SHR	Aged	SA7	80.0	Wistar	Aged	WA7	74.0
SHR	Aged	SA8	80.0	Wistar	Aged	WA8	74.0
SHR	Aged	SA9	80.0	-	-	-	-
SHR	Aged	SA10	80.0	-	-	-	-
SHR	Old	SO1	264.0	Wistar	Old	WO1	265.0
SHR	Old	SO2	288.0	Wistar	Old	WO2	261.0
SHR	Old	SO3	330.0	Wistar	Old	WO3	225.0
SHR	Old	SO4	330.0	Wistar	Old	WO4	251.0
SHR	Old	SO5	330.0	Wistar	Old	WO5	264.0
SHR	Old	SO6	330.0	Wistar	Old	WO6	247.0
SHR	Old	SO7	330.0	-	-	-	-
SHR	Old	SO8	251.0	-	-	-	-

### 2.2 Sample collection and processing

Animals were deeply anaesthetized with isoflurane (5% in O_2_, 1 L/min via inhalation) until loss of paw withdrawal reflex, and then given heparin intraperitoneally (350 UI, Pfizer, Australia). Maintaining isoflurane anaesthesia, animals were injected with a lethal dose of pentobarbital sodium (300 mg/kg; Euthatal) intraperitoneally, and the onset of death was confirmed by the absence of withdrawal reflex to a hind paw noxious pinch and respiratory cessation. Animals were intracardially perfused with 300 mL of ice-cold phosphate-buffered saline (PBS; pH 7.4) and then 300 mL of ice-cold 4% (w/v) paraformaldehyde solution (PFA; Sigma-Aldrich) in PBS at a constant rate (∼30 mL/min) using a peristaltic pump.

Animals were then transferred to a surgical area, and common carotid artery bifurcations were accessed via the anterior midline neck incision. The common carotid artery bifurcation was gently separated from the surrounding tissues and cut above the hypoglossal nerve, transversing the bifurcation to ascertain intact CB collection. Dissected bifurcations were quickly rinsed in ice-cold PBS to remove any remaining blood and transferred to chilled 4% (w/v) PFA solution. Samples were fixed overnight at 4°C before being transferred to 30% sucrose (S9378; Sigma-Aldrich) solution in PBS and kept for 24 h at 4°C. Following tissue fixation and cryoprotection, tissue samples were embedded in OCT compound (Shandon™ Cryomatrix™, Thermo Fisher Scientific), frozen on dry ice, and stored at −80°C until processed.

Common carotid artery bifurcations were cryosectioned to capture the entire CB in transverse orientation in 20-μm-thick serial sections from the rostral-to-caudal direction.

This was achieved by inspecting for the presence of the CB tissue under a benchtop bright-field microscope, where a ‘pilot’ section was examined every 100 μm after staining with 0.1% (w/v) toluidine blue (89640; Sigma-Aldrich). Once the first glomus cell clusters were identified, all subsequent tissue sections were mounted on SuperFrost Plus™ (10149870; Thermo Scientific) microscopy slides. The collection was stopped once the absence of the CB was confirmed by contrast illumination under the benchtop bright-field microscope. During the collection, sections were placed on slides in chronological order, representing the rostral-to-caudal progression. If a section was lost during cutting, an empty space corresponding to the missing section was introduced on the microscopy slide, and a note was taken of the occurrence, preserving the chronological order of rostral-to-caudal progression.

**Figure 2:**
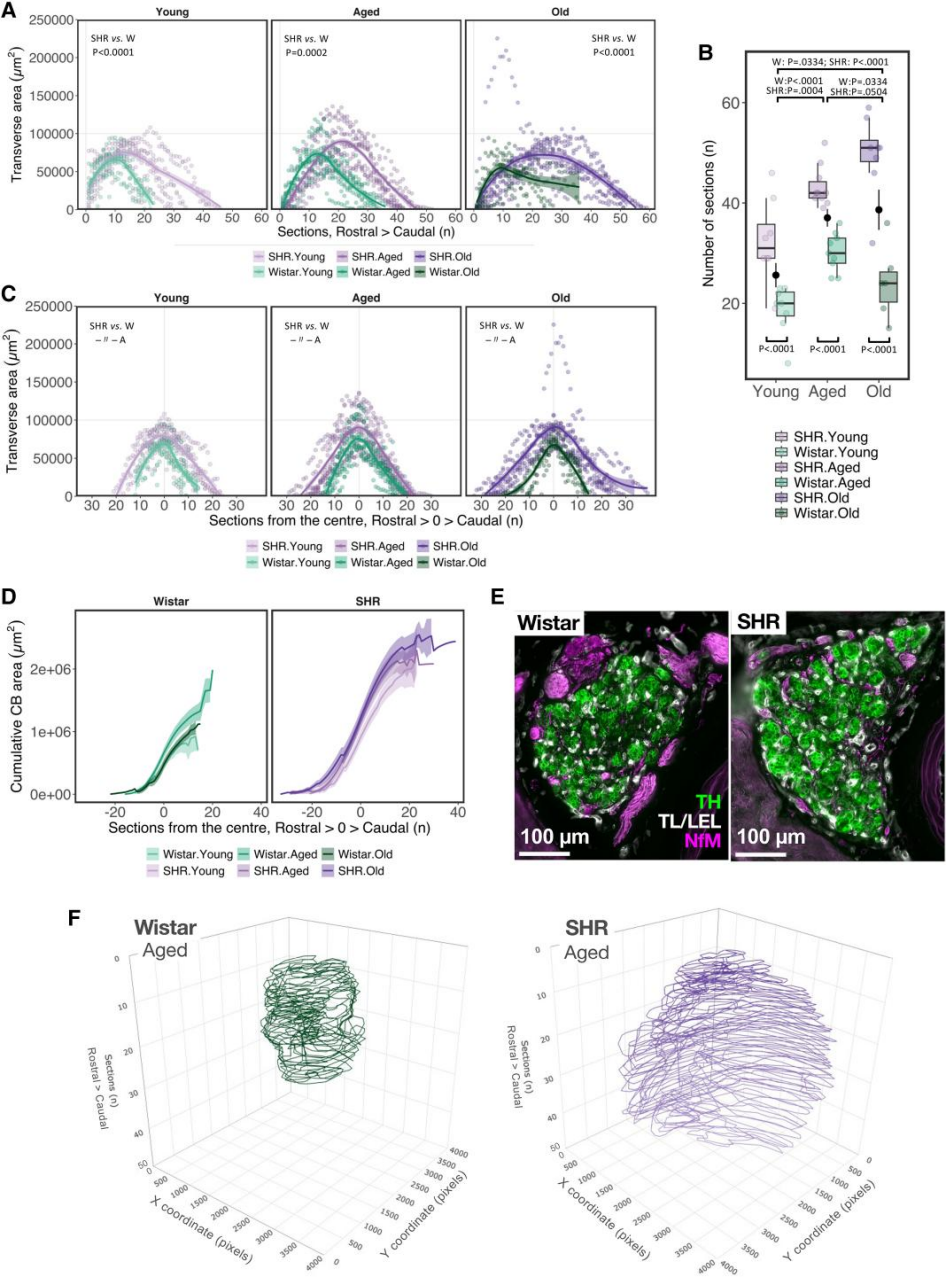
Carotid body hypertrophy in SHR precedes the onset of hypertension. (*A*) Transverse CB area plotted for each serial section along the rostral–caudal axis. (*B*) CB size in the sagittal axis is measured as the number of 20 µm sections spanning the CB. Coloured dots indicate observed values for each animal. Black dots represent the mean value across strains within each age group. Boxplots represent strain within each age group. (*C*) Data from panel *A* replotted so that the largest CB transverse area in each animal is set as the centre (0) along the rostral–caudal axis. (*D*) Cumulative CB area where the largest transverse area in each animal is assigned as the centre (0) along the rostral–caudal axis. (*E*) Representative image of CB size and shape in the transverse plane. Example selected from young SHR and Wistar rats, showing the section with the widest (0) area closest to the group average. TH—tyrosine hydroxylase (green). NfM—neurofilament-M (magenta). TL/LEL—tomato (*Lycopersicon esculentum*) lectin (white). (*F*) A representative 3D render of CB size and shape in aged SHR and Wistar rats. Area outlines are centred on the *xy* coordinate plane and manually adjusted for radial alignment. *n* = 1. For *A* and *C*, lines represent smoothed trends calculated using LOESS estimation. In *A*, *C*, and *D*, shaded areas represent a 95% confidence interval. Sample size—Young: W, *n* = 7, *N* = 150; SHR, *n* = 8, *N* = 260; Aged: W, *n* = 8, *N* = 272; SHR, *n* = 10, *N* = 432; Old: W, *n* = 6, *N* = 145; SHR, *n* = 8, *N* = 396 (*n*—biological replicates, *N*—observation). Hypothesis testing was performed on data summarized in *A* and *B* using a generalized additive model (GAM) with log-transformed data fitted to a Gaussian distribution. For *C*, a generalized linear model (GLM) was fitted to a Poisson distribution. *P*-values were derived from a *post-hoc* estimated marginal means test for specified comparison groups (*n* = 9) using false discovery rate (FDR) multiple comparison correction.

During image acquisition, the rostral–caudal section counter was adjusted by the number of missing sections at the corresponding position to maintain the correct transverse area–sagittal axis relationship. No sections were lost during multiplex fluorescence labelling of tissue. In rare cases where a micrograph of the CB could not be acquired due to a bubble in the mounting medium, a micrograph was taken, but no measurement was recorded for that section. The total number of sections included in each analysis is reported in the corresponding figure legends. Throughout the manuscript, data are presented with the largest transverse area in each animal assigned as the centre (0) along the axis (e.g. *Figure 2C*). This allowed for accurate representation of lateral (rostral–caudal) sections lost during tissue processing. All analyses presented herein were conducted on the right CB, examined unilaterally.

### 2.3 Multiplex fluorescence labelling

A hydrophobic barrier was drawn around the sections using the ImmEdge® PAP Pen (H-4000; Vector Laboratories), and slides were rinsed in PBS to wash off the OCT. Subsequently, sections were incubated in permeabilization-blocking solution containing 5% normal donkey serum (v/v), 5% normal goat serum (v/v), and 0.3% Triton X-100 (v/v) solution in PBS for 2 h at room temperature. Sections were rinsed in PBS and incubated in primary antisera solution in 5% normal serum at 4°C overnight. Primary antibodies were rabbit monoclonal anti-tyrosine hydroxylase (TH; 1:500; AB152; Merck; RRID: AB_390204) and mouse monoclonal anti-neurofilament 160 kDa (NF) Antibody (1:125; 2H3; DHSB; RRID: AB_531793). Sections were rinsed 3 × 10 min in PBS and incubated in the secondary antisera solution containing secondary antibodies, *Lycopersicon esculentum* (tomato) lectin conjugated to DyLight®-647 (1:250; DL-1178-1; Vector Laboratories) and DAPI (1:10 000; D9542; Sigma-Aldrich) for 2 h at room temperature. Secondary antibodies used in the study were Donkey anti-mouse IgG AF594 (1:500; A21203; Invitrogen) and Goat anti-rabbit IgG AF488 (1:500; A11008; Invitrogen). Lastly, sections were rinsed 3 × 10 min in PBS and mounted in ProLong™ Diamond Antifade Mountant (P36970; Invitrogen) under No. 1.5H precision cover glasses (0107242; Marienfield Superior) and left to set overnight, protected from light at room temperature. No primary antibody control immunolabelling was performed for each antibody used in the study during pilot optimization experiments, and consistently resulted in an absence of immunofluorescence. As antibodies used in the study are well-characterized (RRID: AB_390204, AB_531793) and target distinct, identifiable cellular compartments within the CB (*Figure 5A*), no further validation of antibody specificity was performed.

### 2.4 Widefield fluorescence microscopy and image analysis

Prepared slides were imaged at the University of Auckland, Biomedical Imaging Research Unit, using an Olympus SLIDEVIEW VS200 ASW 4.1 imaging system equipped with a Hamamatsu ORCA-Flash4.0 V3 Digital CMOS camera. Multichannel images were acquired using fluorescence filter sets for DAPI (Ex: 365/20 nm; Em: 450/40 nm), FITC (490/20 nm; 525/36 nm), Cy3 (555/25 nm; 590/20 nm), and Cy5 (645/30 nm; 700/50 nm) powered by XYLIS XT720S LED illumination. Images were acquired using an Olympus UPLXAPO 40x/0.95 air objective. Multiple fields of view were tiled together to capture the entire CB area defined manually from an overview image. All images were captured as nine optical sections (*z*-stack), spaced 0.42 µm apart and centred on a manually set focal point for each section. Exposure was kept constant across all images. The resulting images were exported as 16-bit. TIFF and processed using ImageJ/Fiji software.

Images were flattened as a maximum intensity projection. Each channel was then deconvolved using the Richardson–Lucy algorithm for 25 iterations using DeconvolutionLab2.^23^ Point spread function (PSF) used for deconvolution was generated using the Born and Wolf 3D optical model using PSF Generator.^24^ In resulting multichannel images, CB was selected manually in each image to outline the perimeter of the organ, primarily based on TH-immunoreactivity.

The area of the TH positive signal was defined using automatic thresholding with the default settings in ImageJ/Fiji.^25^ Areas containing tomato lectin (TL)/LEL and NF positive signal were obtained using a custom artificial neural network pixel classifier trained on a set of representative training images from all groups using QuPath (v0.5.1).^26^ For all channels, the area within the manually defined CB boundaries was measured and presented in µm^2^. Density was calculated as a percentage of the reference area containing the signal of interest. Area masks for each process were inspected for extreme values; if such values occurred (e.g. NF^+^ area >60% of total CB area due to high background fluorescence), the original images were manually reviewed. Images affected by processing artefacts, e.g. out of focus signal, were discarded. The number of images (*N*) included in each analysis is indicated in the figure legends.

The proportion of NF^+^ signal co-labelled with TH was calculated by applying the NF^+^ area mask onto the TH channel image within the predefined CB area. The TH^+^ area was then quantified using automatic thresholding in ImageJ/Fiji, as described above.

Nuclei identification was done by applying the ‘dsb2018_heavy_augment.pb’ model to the DAPI channel using StarDist^27^ in QuPath. For nuclei quantification, a separate threshold-based classifier was applied to define the area corresponding to TH^+^ chemosensory clusters. This was done to loosely isolate the chemosensory cluster regions, ensuring the inclusion of nuclear spaces within the cluster while excluding TH^+^ immunoreactive fibres outside the cluster (see Supplementary material online, *Figure S1*).

Cumulative area (*Figure 2D*) was calculated by sequentially summing the transverse area of each section with that of the next (*n* + 1) for each replicate.

3D rendering of CB size and shape was performed by first setting canvas dimensions to be equal across all Wistar and SHR images used. The regions of interest corresponding to the CB area in each image were then loaded and centred on the scaled canvas image. Polygon coordinates were extracted from the scaled images for each section using the ROI manager in ImageJ/Fiji. The area outlines are centred on the *xy* coordinate plane as captured during image acquisition and manually adjusted for radial alignment. 3D line plots (*Figure 2F*) were rendered using Plotly.^28^

### 2.5 Data visualization

Boxplots represent the median, lower, and upper hinges correspond to the first and third quartiles (the 25th and 75th percentiles), whisker extends from the hinge to the largest (or lowest value) value no further than 1.5 * IQR from the hinge. Black dots represent mean ± SE for both strains for each age group. Coloured dots represent observed data where values from each replicate are aligned in vertical lines. In scatter plots, lines represent a smoothing function based on the ‘loess’ (locally estimated scatterplot smoothing) method selected as per the ‘ggplot2’ default functionality for <1000 observations. Shaded areas represent a 95% confidence interval. Coloured dots represent observed data. For data measured in percentages, observed values in µm^2^ were expressed as a proportion compared with the reference signal (% of area occupied).

### 2.6 Statistical analysis

**Figure 3:**
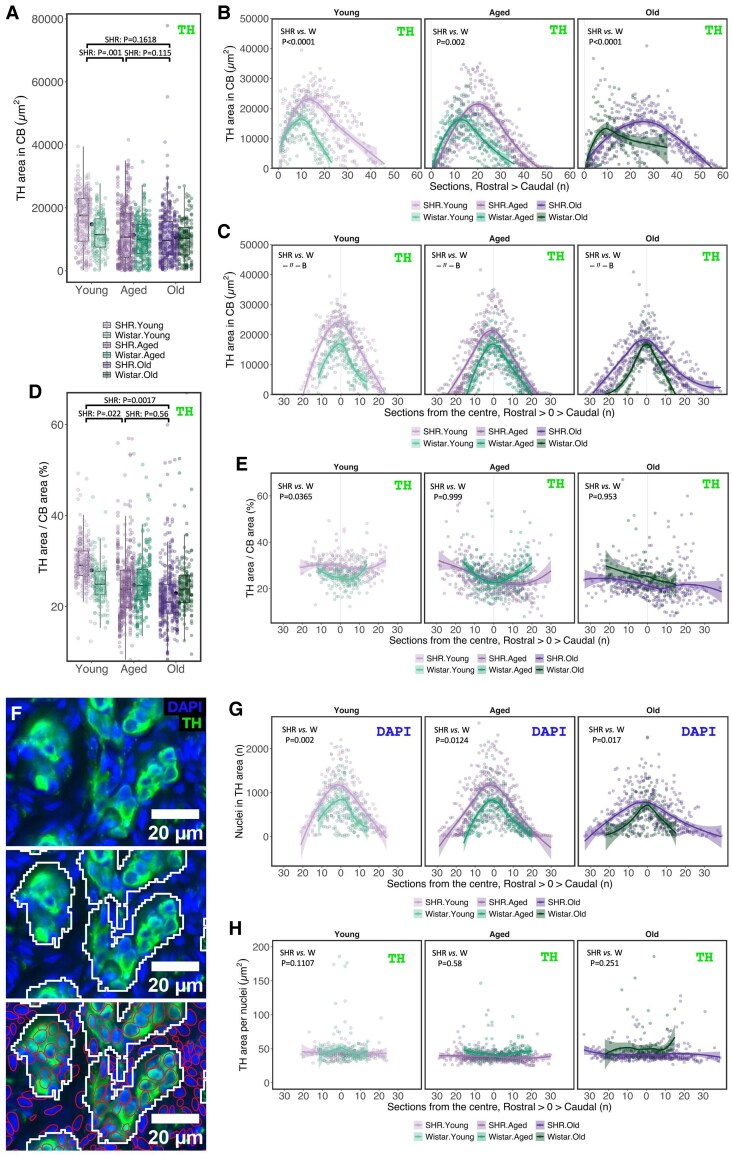
Carotid body hypertrophy in hypertension is driven by chemosensory cell hyperplasia. (*A*) TH-immunoreactive (TH^+^) area in the CB. (*B*) Data from panel *A* replotted to show TH^+^ area for each serial section along the rostral–caudal axis. (*C*) Data from panel *A* replotted to show TH^+^ area where the largest CB transverse area in each animal is set as the centre (0) along the rostral–caudal axis. (*D*, *E*) Proportion of CB area immunoreactive for TH summarized for each group (*D*) and plotted relative to the largest transverse area in each animal set as the centre (0) along the rostral–caudal axis (*E*). (*F*) Representative image showing nuclei quantification in chemosensory (glomus) cell clusters. The TH-immunoreactive area was defined using a threshold-based pixel classifier in QuPath, providing a rough outline of the chemosensory clusters. DAPI-stained nuclei were counted using automated StarDist^27^ object segmentation. (*G*) Number of nuclei within the TH^+^ area in each section. Data are presented where the section with the largest CB transverse area in each animal was set as the centre (0) along the rostral–caudal axis. (*H*) Calculated TH^+^ area per nuclei in each section. Data are presented where the section with the largest CB transverse area in each animal was set as the centre (0) along the rostral–caudal axis. Sample size—Young: W, *n* = 7, *N* = 150; SHR, *n* = 8, *N* = 260; Aged: W, *n* = 8, *N* = 272; SHR, *n* = 10, *N* = 432; Old: W, *n* = 6, *N* = 145; SHR, *n* = 8, *N* = 396 (*n*—biological replicates, *N*—observation). For *A* and *D*, observed values (coloured dots) for each biological replicate (animal) are aligned vertically. Black dots represent the mean value across strains within each age group. Boxplots represent strain within each age group. For *B*, *C*, *E*, *G*, and *H*, lines represent smoothed trends calculated using LOESS estimation, and shaded areas represent the 95% confidence interval. Hypothesis testing was performed using a generalized additive model (GAM) with log-transformed data fitted to a Gaussian distribution for *A*–*E*, *H*, and a Poisson distribution for *G*. *P*-values were derived from a *post-hoc* estimated marginal means test for specified comparison groups (*n* = 9) using false discovery rate (FDR) multiple comparison correction.

Hypothesis testing was performed using mixed regression models in R. For area data (µm^2^), a generalized additive model (GAM) was fitted to log-transformed data with a Gaussian distribution, employing the mgcv package (v1.8-41).^29^ The categorical variables of ‘Strain’ (Wistar (*reference group*), SHR) and ‘Age_group’ (Young (*reference group*), Aged, Old) were included as fixed effects. A smooth function was applied to our predictor ‘Section’ to account for its non-linear relationship with the dependent variable. The latter was estimated for each ‘Strain’. Lastly, ‘Sample_ID’ was included as a level-2 clustering variable to estimate random effect variances across all animals’ intercepts (i.e. accounting for nested data). For the number of sections (*Figure 2C*), we fitted a generalized linear model (GLM) with Poisson distribution, employing the R base ‘stats’ package. For nuclei count (*Figure 3G*), a GAM was fitted to a Poisson distribution. For density data (presented as %), analysis was performed on observed area values (µm^2^) as described above for GAM, but a tensor product smooth function for two variables, e.g. ‘te(Section, log(CB_area_um2), by = Strain)’, was used in place of the smooth function. The model fit was assessed by comparing the percentage of deviance explained, Akaike Information Criterion, plotting the fitted values against the observed data, inspecting the residuals vs. fitted values scatterplot, and the residuals histogram. *Post-hoc* pairwise comparisons were performed using estimated marginal means for specified comparisons (*n* = 9), using the emmeans package (v1.10.2). False discovery rate (FDR) correction was applied to account for multiple comparisons. Statistical significance was assumed if *P* < 0.05.

## 3. Results

### 3.1 CB hypertrophy precedes the onset of hypertension in the SHR

To determine if hypertension is associated with changes in CB composition, we first examined differences in CB size between Wistar and SHR.

To illustrate this, we plotted the transverse area for each serial section collected in sequence in the rostral–caudal direction (*Figure 2A*). Compared with Wistar controls, SHR had approximately 68.9% larger transverse CB area (GAM_Strain_: Est = 1.689, CI 95%: 1.16–2.45, *P* = 6.13E-03) that was consistently greater across all age groups (*Figure 2A*). Next, we assessed differences along the sagittal axis, measured as the number of sections (20 µm) spanning the organ. On average, the CB in SHR spanned 73.3% more sections compared with Wistar counterparts (SHR median—42 sections (∼840 µm) vs. Wistar—24 (∼480 µm); GLM_Strain_: Est = 1.733, CI 95%: 1.42–2.12, *P* = 8.11E-08) (*Figure 2B*). Age had a significant effect on the CB size along the sagittal axis, with the number of sections increasing from ‘young’ to ‘aged’ in both Wistars and SHR (GLM_Age_group–Aged_: Est = 1.62, CI 95%: 1.32–1.96, *P* = 2.68E-06), whereas from ‘aged’ to ‘old’, CB size increased further in SHR (P.adj_FDR_ = 0.0504) and was smaller in Wistars (P.adj_FDR_ = 0.0334) (*Figure 2B*).

To reflect sagittal axis variation for a more accurate assessment of size differences in the transverse plane, we assigned the largest transverse area in each animal as the centre (0) along the rostral–caudal axis (*Figure 2C*). This revealed that the transverse CB area in SHR was consistently greater when matched for three-dimensional variation along the sagittal plane. Notably, age had no effect on the CB area in Wistar, whilst in SHR, the transverse area was significantly larger in ‘Aged’ compared with the ‘Old’ group (P.adj_FDR_ = 0.0249); however, the effect size of this change appeared small considering the average values (*Figure 2A* and *C*). To better gauge CB size differences in three dimensions, we calculated the cumulative CB area (*Figure 2D*). Combining sagittal elongation (*z*) and transverse expansion (*xy*) concurred with a larger CB size in ‘old’ compared with ‘aged’ SHR, and a reduction from ‘aged’ to ‘old’ Wistar rats (*Figure 2D*).

Qualitatively, we also observed changes in CB shape associated with hypertension (*Figure 2E*). While the transverse area in normotensive Wistar was predominantly oval, in SHR, the CB appeared semicircular, extending along the outer perimeter of the internal carotid artery. In this regard, we observed no differences between age groups. To provide a better sense of scale regarding the CB size difference between Wistar and SHR strains, we generated a three-dimensional rendering of CB area outlines from a representative aged animal (*Figure 2F*, see Supplementary material online, *Files S1* and *S2*). These analyses showed that hypertension is associated with structural alterations and doubling of CB volume in 4–6-week-old SHR without established hypertension.

### 3.2 CB hypertrophy is driven by chemosensory cell hyperplasia in the SHR

Next, we examined whether hypertension alters TH-immunoreactivity—a marker of chemosensory (glomus) cells in the CB. Compared with Wistar controls, SHR carotid bodies had approximately 91.3% larger TH-immunoreactive (TH^+^) area (GAM_Strain_: Est = 1.913, CI 95%: 1.32–2.76, *P* = 5.81E-04) that was consistently larger across age groups (*Figure 3A–C*). Age reduced TH^+^ area in aged compared with young SHR rats (P.adj_FDR_ = 0.0011). Next, we tested if the increase in TH-immunoreactivity was proportional to the increase in CB size by expressing the data as a percentage of the CB area positive for TH (*Figure 3D*). This revealed strain to have a significant effect on TH density (GAM_Strain_: Est = 2.35, CI 95%: 1.16–2.45, *P* = 0.0174) that stemmed from an increase in the young SHRs only (P.adj_FDR_ = 0.036) (*Figure 3E*). Interestingly, age had a strain-dependent effect on TH density, where it was unaffected in aging Wistars and progressively decreased in aging SHR (*Figure 3D*). To further explore whether TH expansion in the SHR was due to chemosensory cell hypertrophy or hyperplasia, we quantified the DAPI-stained nuclei within the TH-immunoreactive area (*Figure 3F*). The TH^+^ area in the SHR had a greater number of nuclei across all age groups (*Figure 3G*). However, the change in cell count was proportional to the increase in TH-immunoreactive area (*Figure 3H*). Collectively, our data show that the hypertrophy of the CB in the SHR is associated with chemosensory glomus cell hyperplasia.

### 3.3 CB hypertrophy in SHR is associated with increased angiogenesis

**Figure 4:**
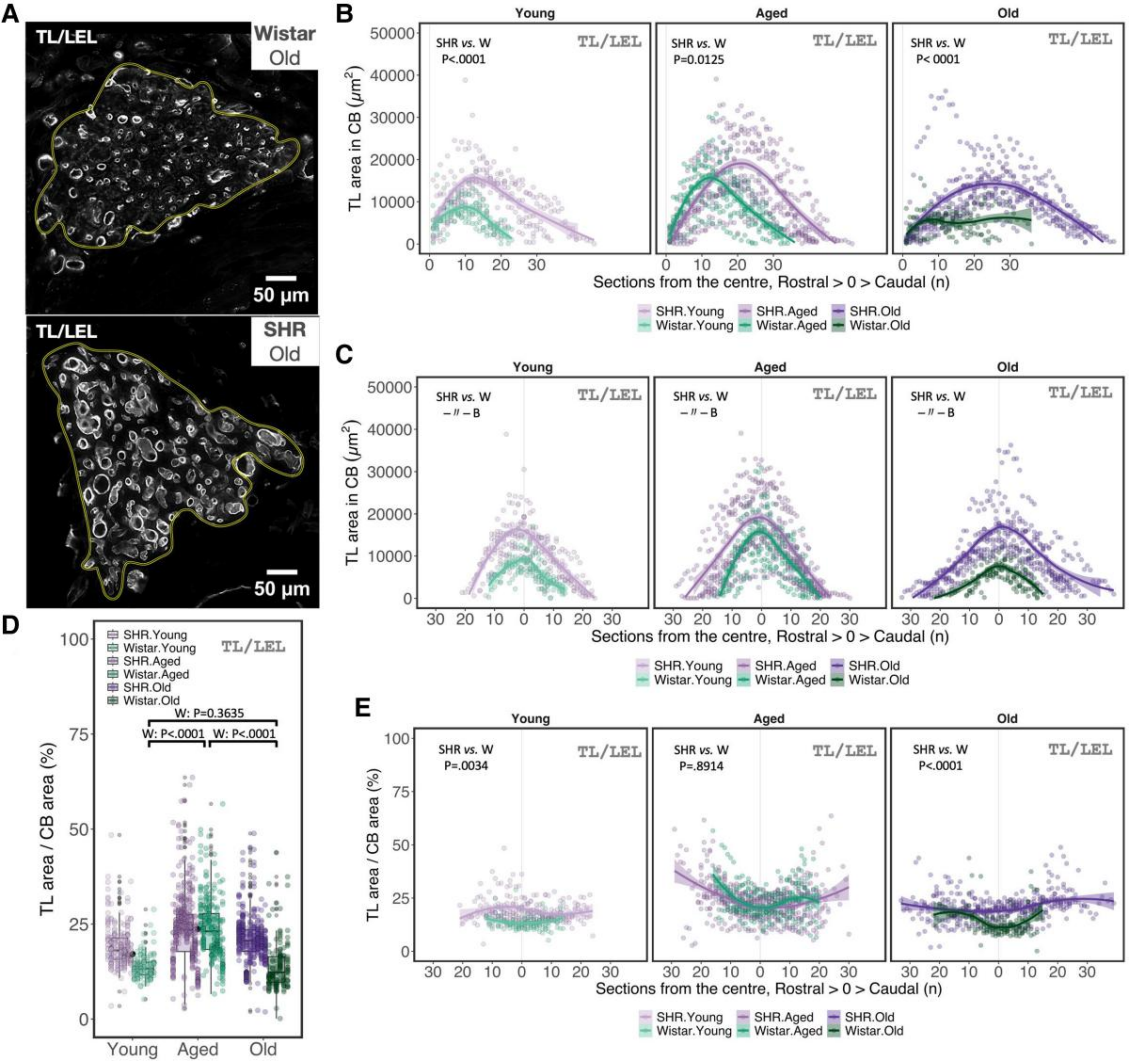
Increased carotid body vascularization in SHR. (*A*) Representative image of the capillary network perfusing the CB. The yellow line outlines the CB area. TL/LEL—tomato (*Lycopersicon esculentum*) lectin (white). (*B*, *C*) Tomato lectin-positive area in the CB plotted for each serial section along the rostral–caudal axis (*B*) and relative to the largest transverse area in each animal set as the centre (0) along the rostral–caudal axis (*C*). (*D*, *E*) Proportion of CB area labelled by TL/LEL summarized for each group (*D*) and plotted relative to the largest transverse area in each animal set as the centre (0) along the rostral–caudal axis (*E*). In *D*, observed values (coloured dots) for each biological replicate (animal) are aligned vertically. Black dots represent the mean value across strains within each age group. Boxplots represent strain within each age group. For *B*, *C*, and *E*, lines represent smoothed trends calculated using LOESS estimation, and shaded areas represent the 95% confidence interval. Sample size—Young: W, *n* = 7, *N* = 142; SHR, *n* = 8, *N* = 238; Aged: W, *n* = 8, *N* = 271; SHR, *n* = 10, *N* = 428; Old: W, *n* = 6, *N* = 144; SHR, *n* = 8, *N* = 396 (*n*—biological replicates, *N*—observation). Hypothesis testing was performed using a generalized additive model (GAM) with log-transformed data fitted to a Gaussian distribution. *P*-values were derived from a *post-hoc* estimated marginal means test for specified comparison groups (*n* = 9) using false discovery rate (FDR) multiple comparison correction.

Next, we investigated if hypertension leads to vascular remodelling within the CB (*Figure 4A*). Once again, SHR exhibited the increased area of TL/LEL signal (GAM_Strain_: Est = 3.77, CI 95%: 1.83–7.76, *P* = 3.1E-04) that was consistently higher in all age groups (*Figure 4B* and *C*). Vascular area in the SHR remained constant across age groups (*P* > 0.05). While in Wistars, TL/LEL^+^ area was significantly higher in aged compared with the young and old animals (Young vs. Aged: P.adj_FDR_ = 0.0195; Aged vs. Old: P.adj_FDR_ = 0.0029). Comparing the vascular density (proportion of the CB area positive for TL/LEL) revealed that it was increased in young and old SHR compared with age-matched Wistar (Young: P.adj_FDR_ = 0.0034; Aged: P.adj_FDR_ = 0.891; Old: P.adj_FDR_ < 0.0001) (*Figure 4E*). Interestingly, vascular density in the SHR remained constant across age groups, whereas in Wistars, it paralleled that observed for absolute TL/LEL^+^ area and was increased in the aged animals compared with young and old groups (*Figure 4D*). Collectively, these data show that SHR exhibit increased vascularization of the CB, characterized by increased total vessel area and vascular density.

### 3.4 Changes in chemoafferent innervation and nerve fibre composition in the SHR

**Figure 5:**
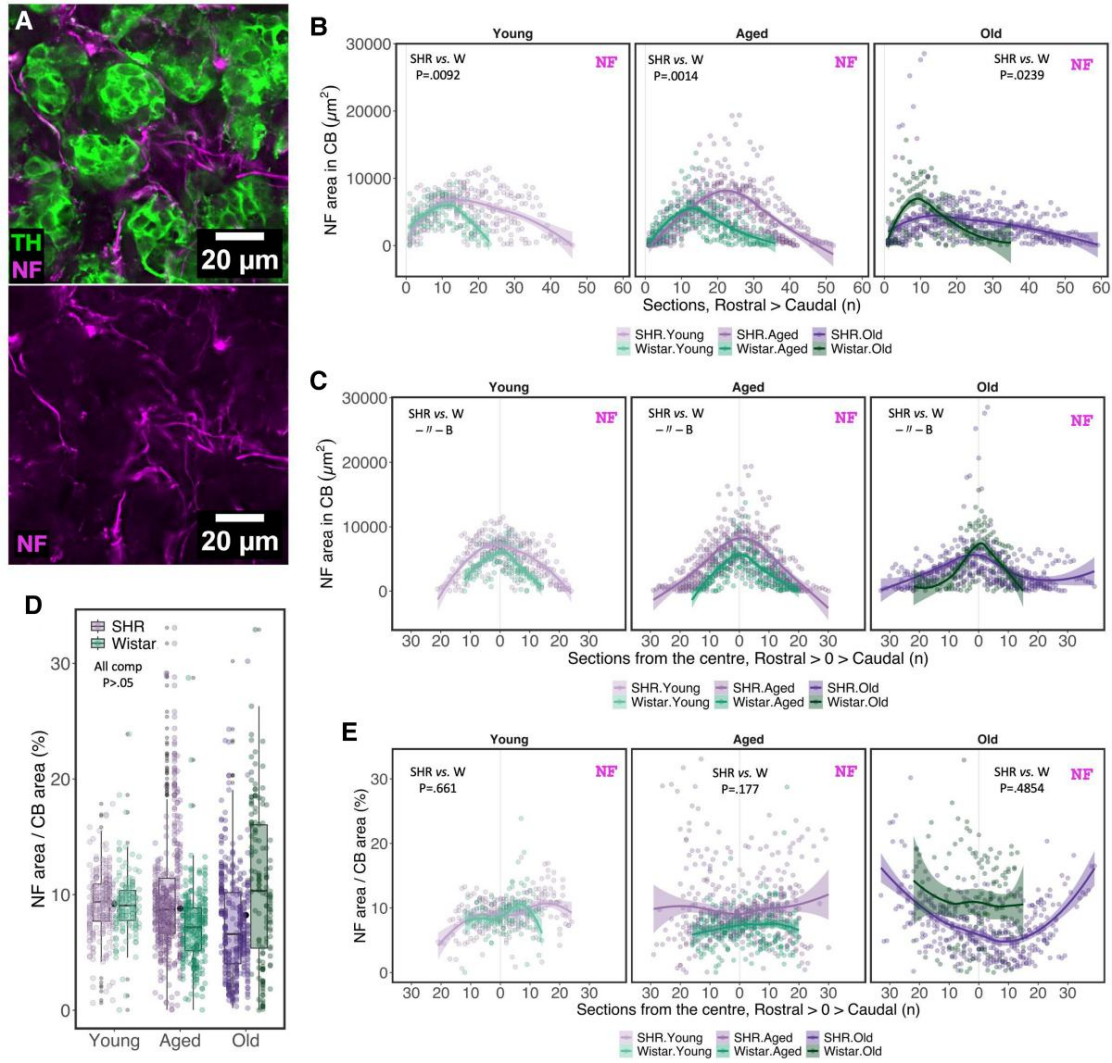
Altered carotid body innervation in SHR. (*A*) Representative image of 160 kDa neurofilament-M (NF) immunolabelling (magenta) in the CB. TH—tyrosine hydroxylase (green). (*B*, *C*) NF-immunoreactive area in the CB plotted for each serial section along the rostral–caudal axis (*B*) and relative to the largest transverse area in each animal set as the centre (0) along the rostral–caudal axis (*C*). (*D*, *E*) Proportion of CB area immunoreactive for NF summarized for each group (*D*) and plotted relative to the largest transverse area in each animal set as the centre (0) along the rostral–caudal axis (*E*). In *D*, observed values (coloured dots) for each biological replicate (animal) are aligned vertically. Black dots represent the mean value across strains within each age group. Boxplots represent strain within each age group. For *B*, *C*, and *E*, lines represent smoothed trends calculated using LOESS estimation, and shaded areas represent the 95% confidence interval. Sample size—Young: W, *n* = 7, *N* = 150; SHR, *n* = 8, *N* = 238; Aged: W, *n* = 8, *N* = 270; SHR, *n* = 10, *N* = 430; Old: W, *n* = 6, *N* = 142; SHR, *n* = 8, *N* = 396 (*n*—biological replicates, *N*—observation). Hypothesis testing was performed using a generalized additive model (GAM) with log-transformed data fitted to a Gaussian distribution. *P*-values were derived from a *post-hoc* estimated marginal means test for specified comparison groups (*n* = 9) using false discovery rate (FDR) multiple comparison correction.

We examined a neuron-specific intermediate (160 kDa) neurofilament (NF) protein as a marker of CB innervation (*Figure 5A*). After accounting for inter-animal and section number-related variance, the area positively labelled for neurofilament (NF^+^) was significantly higher in SHR compared with age-matched Wistars (*Figure 5B*). Age had no significant effect on the NF^+^ area in the CB in either strain (GAM_Age_group–Aged_: Est = 1.09, CI 95%: 0.59–1.99, *P* = 0.786; GAM_Age_group–Old_: Est = 0.92, CI 95%: 0.47–1.78, *P* = 0.81) (*Figure 5C*). However, a downward trend was observed in aging animals (*Figure 5C*). After adjusting for increased CB area in the SHR, we found no difference in innervation density (proportion of CB area positive for NF) between normotensive and hypertensive animals in either age group (*Figure 5D* and *E*). Our data suggests that the increase in NF^+^ area represents an increase in CB innervation in the SHR, where innervation density remains proportional to CB size.

**Figure 6:**
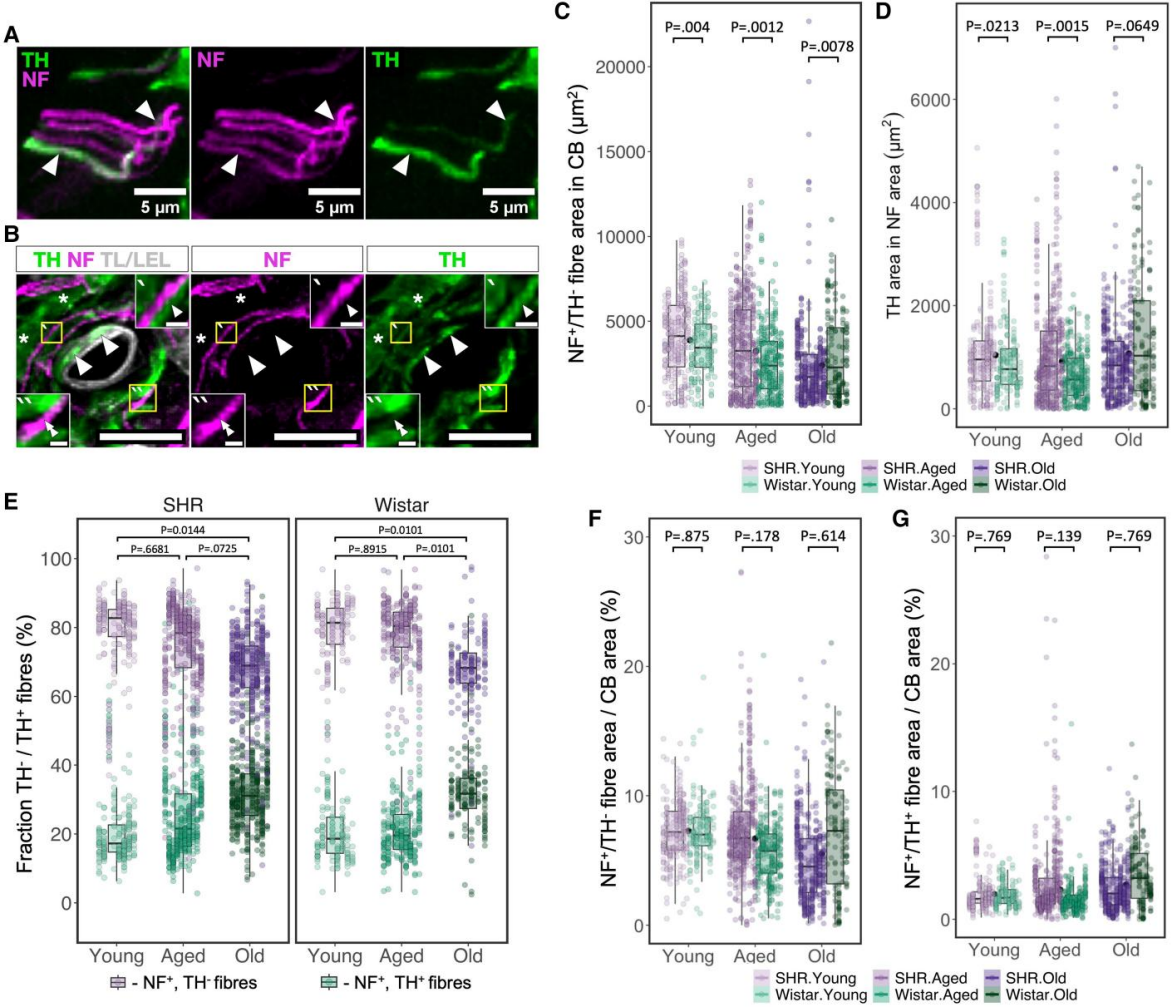
Age-dependent remodelling of chemoafferent innervation of the carotid body. (*A*) Two types of nerve fibres (NF^+^, TH^+^ and NF^+^, TH^−^) innervating the CB. Arrowheads mark NF^+^, TH^+^ fibres. (*B*) Sympathetic motor terminals (NF^−^, TH^+^) in close association with CB vasculature (arrowheads). Double arrowheads mark NF^+^, TH^−^ fibres. Asterisk mark TH^+^ chemosensory cells. TH—tyrosine hydroxylase (green). NfM—neurofilament-M (magenta). TL/LEL—tomato (*Lycopersicon esculentum*) lectin (white). Scale bar—10 μm. Scale bar (insets)—1 μm. C—Area of NF^+^, TH^−^ fibres and (*D*) NF^+^, TH^+^ fibres in the CB. (*E*) Proportion of NF-immunoreactive area corresponding to NF^+^, TH^−^/NF^+^, TH^+^ fibres. (*F*, *G*) Proportion of the CB area occupied by NF^+^, TH^−^ (*F*), and motor (*G*) fibres. In *C*–*G*, observed values (coloured dots) for each biological replicate (animal) are aligned vertically. In *C*, *D*, *F*, and *G*, black dots represent the mean value across strains within each age group. Boxplots represent strain within each age group. In *E*, each section is represented by two values comprising a total of 100%: (i) percentage of NF^+^ area co-localizing with TH (NF^+^, TH^+^) and (ii) remaining NF^+^ area (NF^+^, TH^−^). Sample size—Young: W, *n* = 7, *N* = 149; SHR, *n* = 8, *N* = 237; Aged: W, *n* = 8, *N* = 269; SHR, *n* = 10, *N* = 430; Old: W, *n* = 6, *N* = 142; SHR, *n* = 8, *N* = 396 (*n*—biological replicates, *N*—observation). Hypothesis testing was performed using a generalized additive model (GAM) with log-transformed data fitted to a Gaussian distribution. *P*-values were derived from a *post-hoc* estimated marginal means test for specified comparison groups (*n* = 9) using false discovery rate (FDR) multiple comparison correction.

To assess whether the net increase in CB innervation is associated with changes in nerve fibre composition, we examined NF^+^/TH^−^ and NF^+^/TH^+^ fibres based on TH-immunoreactivity (*Figure 6A*). Notably, this analysis was limited to axonal branches containing intermediate (160 kDa) neurofilaments as the NF^+^ signal did not label either chemosensory (*Figure 5A*) or adrenergic postganglionic end terminals (*Figure 6B*). We found the absolute area of NF^+^/TH^−^ fibres to be consistently higher in the SHR across all age groups (*Figure 6C*), whereas the NF area co-labelled with TH (NF^+^/TH^+^) was greater in young and aged SHR but not in old animals compared with age-matched Wistars (*Figure 6D*). Adjusting for differences in absolute NF area and expressing the data as a proportion of all NF-immunoreactive fibres co-labelled with TH revealed an age-dependent remodelling of CB innervation (*Figure 6E*). Here, old age was associated with a strain-independent decrease in NF^+^/TH^−^ fibres coupled to an inverse rise in the NF^+^/TH^+^ fibre area (GAM_Age_group–Old_: Est = 1.62, CI 95%: 1.2–2.2, *P* = 1.58E-03). After adjusting for changes in CB size, we found no strain differences in the density of NF^+^/TH^−^ and NF^+^/TH^+^ innervation (*Figure 6F* and *G*). Together, these data suggest that the increase in CB innervation in SHR is associated with an age-dependent shift in nerve fibre composition, characterized by a reduction in NF^+^/TH^−^ fibres, while NF^+^/TH^+^ fibres remain unchanged, leading to an increased proportion in aging CB.

### 3.5 CB hypertrophy in the SHR is not linked to changes in the extracellular, extravascular space

**Figure 7:**
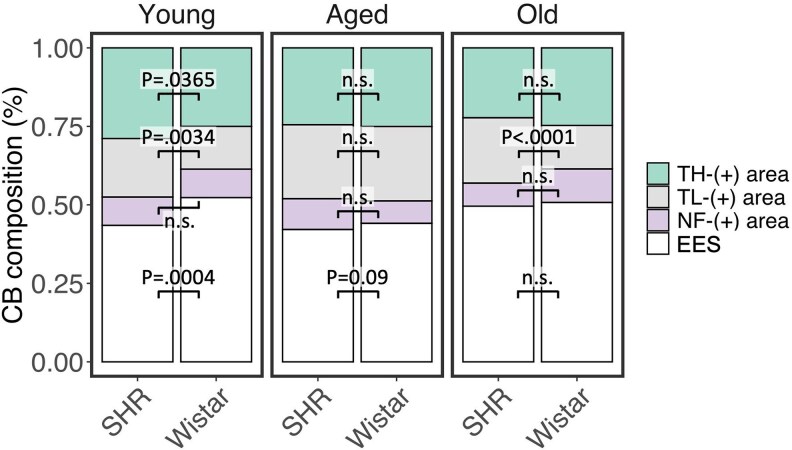
Extracellular, extravascular space (EES) dynamics reflect but do not drive changes in CB composition. EES proportion for each section was calculated as the CB transverse area (*Figure 2A*) minus the percentage areas of chemosensory (TH^+^, *Figure 3D*), vascular (TL^+^, *Figure 4D*), and neural (NF^+^, *Figure 5D*) components. Sample size—Young: W, *n* = 7, *N* = 142; SHR, *n* = 8, *N* = 238; Aged: W, *n* = 8, *N* = 270; SHR, *n* = 10, *N* = 426; Old: W, *n* = 6, *N* = 141; SHR, *n* = 8, *N* = 396 (*n*—biological replicates, *N*—observation). Hypothesis testing was performed using a generalized additive model (GAM) with log-transformed data fitted to a Gaussian distribution. *P*-values were derived from a *post-hoc* estimated marginal means test for specified comparison groups (*n* = 9) using false discovery rate (FDR) multiple comparison correction.

Lastly, we examined changes to the extracellular, extravascular space (EES) as the proportion of the CB area remaining after subtracting the chemosensory, vascular, and neural compartments. This indicated strain to have a significant effect on EES area (GAM_Strain_: Est = 0.781, CI 95%: 0.68–0.89, *P* = 3.4E-03); however, this effect was mainly explained by divergent age-related differences between strains (*Figure 7*). In Wistar, the EES area was significantly reduced in Aged compared with Young (Young vs. Aged: P.adj_FDR_ = 0.012) and Old (Aged vs. Old: P.adj_FDR_ = 0.026) animals. Whereas in SHR, the EES area was lower in Young and Aged animals compared with that in Old animals (Young vs. Old: P.adj_FDR_ = 0.021; Aged vs. Old: P.adj_FDR_ = 0.012). Collectively, these data suggest that changes in EES are associated with, but do not appear to drive, the increase in CB size in the SHR.

## 4. Discussion

Herewith, we present the most detailed morphometric analysis of the hypertensive CB in the SHR to date. We show that CB hypertrophy in the SHR is driven by hyperplasia in the TH-immunoreactive chemosensory clusters, coinciding with the expansion of the capillary network supplying the organ. Most importantly, we provide novel evidence that CB hypertrophy in the SHR is linked to increased chemoafferent innervation and demonstrate an age-dependent remodelling of nerve fibre composition. This study also offers a standardized methodology for investigating CB composition and innervation from serially sectioned, two-dimensional image data to facilitate comparison across different studies. Lastly, the image data generated has been made publicly available to support the modelling of arterial chemosensory function.

While changes in CB morphology in the SHR have been previously mapped,^12–17^ no clear consensus has emerged regarding the basis of CB hypertrophy in hypertension. Our study aimed to clarify this by simultaneously examining all principal components of the neurovascular interface whilst accounting for variance in the organ’s three-dimensional structure. Increased cell proliferation (hyperplasia) in the CB of SHR has been previously reported;^14,15^ however, subsequent reports found no evidence to support this.^12,16^ Similarly, earlier reports hinted at increased TH abundance in the hypertensive CB,^30^ whereas later studies reported conflicting results.^22,31^ Importantly, none of these studies have directly linked changes in CB cellularity to alterations in TH levels, a marker of chemosensory glomus cells, which is addressed by the present investigation.

The molecular pathways underlying CB hypertrophy in response to hypoxia are well established, with hypoxia-inducible factor 2 alpha (HIF-2α, encoded by *Epas1*) playing a central role.^7,32^ Sustained hypoxia stimulates chemosensory (TH^+^) cell proliferation^7^ that is dependent on *Epas1* expression.^32,33^ Importantly, CB hypertrophy is regulated by the HIF-2α catalytic enzyme prolyl hydroxylase domain 2 (PHD2, encoded by *Egln1*). Deficiency or pharmacological inhibition of PHD2 leads to both chemosensory (TH^+^) cell hyperplasia and angiogenesis.^33–35^ Notably, in our recently published RNA-seq dataset comparing CBs from SHR and WKY rats, we detected no change in *Epas1* expression.^36^ However, PHD2 (*Egln1*) in the SHR was significantly downregulated (fold-change: 0.8, P.adj = 0.026).^36^ These findings are further supported by our later work, which identified significant upregulation of the HIF-1β (*Arnt2*) transcriptional regulatory network in the CB of SHR.^37^

Conflicting reports also exist regarding alterations in CB vascularization in SHR, with some studies noting a reduction in total vascular volume, while others report no significant change compared with normotensive controls.^12,16,17^ Here, we provide strong evidence that total vascular area within the CB expands proportionally with organ size, in addition to increased vascular density in young and old SHR (*Figure 4*). This is supported by RNA-seq data showing upregulation of angiopoietin-2 (*Angpt2*; fold-change: 1.21, P.adj = 0.0047) in the CB of SHR.^36^ This is particularly significant, as the haemodynamic changes resulting from an expanding vascular network may affect the organ’s perfusion and stimulate the HIF-PHD pathway, enhancing arterial chemosensitivity through a positive feedback loop. This is supported by our finding that CB size in the SHR continues to increase with aging (*Figure 2C*). Collectively, these data suggest that altered HIF signalling in the SHR likely mediates the observed chemosensory hyperplasia and angiogenesis linked to CB hypertrophy. Possible mechanisms driving HIF pathway activation in SHR CBs include: (i) Epigenomic dysregulation present from birth, determining CB development and sensitivity,^38^ and/or (ii) a hypothesized state of local hypoxia within the CB microenvironment related to sympathetically mediated arterial vasoconstriction leading to hypoperfusion of the CB.^19,39,40^ This remains the focus of future research aimed at understanding the emergence of arterial chemoreflex sensitization in cardiometabolic disease.

Our data confirm that CB hypertrophy occurs before established hypertension in the SHR, whereas excess sympathoexcitation appears to coincide with the increased arterial chemoreceptor size.^41,42^ This is supported by the fact that pre-hypertensive neonate and juvenile SHR exhibit excess resting sympathetic activity (eSNA) and augmented chemoafferent sensitivity.^41,42^ Systolic blood pressure (SBP) in SHR starts to diverge from normotensive controls at 4 weeks and becomes fully established by 15 weeks postnatally.^43^ These findings support the idea that increased CB size is associated with aberrant chemoafferent signalling, the latter being a principal generator of eSNA and subsequent aetiology of hypertension in the SHR model.^44,45^ This is further evidenced by the lack of any additional reduction in mean arterial pressure following hexamethonium ganglionic blockade in SHR that underwent bilateral CB removal.^44^

Our data in the SHR highlight clinical evidence of CB hypertrophy in cardiometabolic disease.^1–4^ All these investigations had a cross-sectional design and cannot determine whether CB hypertrophy is a cause or a consequence of the disease state. Interestingly, blood pressure is commonly elevated in patients with CB tumours (CBT) compared with control patients.^46^ Whereas CBT resection is associated with a significant reduction in arterial pressure,^46,47^ akin to that observed in preclinical models of pathological CB enlargement.^6,44^

Furthermore, a positive correlation has been observed between SBP, as well as SBP variability, and CB volume in patients with arterial hypertension.^48^

Additional coinciding evidence from human studies shows a positively skewed distribution of hypoxic ventilatory response (HVR) sensitivity,^49^ paralleled by a positively skewed distribution of CB size reported by two independent studies.^50^ Collectively, these data suggest that CB hypertrophy is coupled to increased HVR sensitivity and may serve as an important prognostic marker for the efficacy of therapies targeting the CB.^10,11^ Especially, considering that higher baseline HVR sensitivity in humans is associated with a greater efficacy of therapies targeting the arterial chemoreflex pathway.^10,51^

Here, we also investigated CB innervation through the quantification of neuronal cytoskeletal proteins (*Figures 5* and *6*). The CB receives innervation from three main sources: (i) the petrosal sensory ganglion containing the bodies of arterial chemoafferent neurons, (ii) the superior cervical ganglion (SCG) containing the bodies of postganglionic sympathomotor neurons, and (iii) autonomic neurons residing within the CB.^52,53^ Direct studies on the neurofilament protein composition of petrosal chemoafferents innervating the CB are limited; however, insights can be drawn from vagal afferent innervation of the airways.^54^ In this context, the main axon of each terminal stains positively for medium (160 kDa) and heavy (200 kDa) neurofilament proteins that disappear soon after the axon ramifies into specialized sensory terminals.^55,56^ Similarly, analysis of neurofilament protein composition in cultured SCG sympathetic neurons showed medium (160 kDa) and light (68 kDa) but not heavy (200 kDa) neurofilaments in SCG neurons.^57^ The 160 kDa intermediate-neurofilament was thus selected in the present study to accurately represent both chemoafferent and sympathomotor arms of arterial chemoreceptor innervation. The remaining source of CB innervation is attributed to autonomic ganglion neurons located within the CB (see Supplementary material online, *Figure S3*). These neurons are considered to represent the parasympathetic arm of autonomic control,^53^ and were seldom observed circumferentially in some of the CBs included in the study. However, due to their relatively low numbers, these intra-CB neurons were not considered to significantly contribute (quantitatively) to the CB innervation described in this study (*Figures 5* and *6*).

We employed a reductionist strategy for differentiating between NF^+^/TH^−^ and NF^+^/TH^+^ fibres based on TH expression (*Figure 6A*). A limitation of this approach is that inferring functionality based on neurochemical phenotypes may be restrictive. Despite TH being an established marker of the sympathomotor fibres, retrograde labelling from the CB showed ∼30% of petrosal chemosensory neurons innervating the organ in the rat are TH-immunoreactive.^58^ This figure closely resembles NF^+^/TH^+^ fibre proportion observed in our study (*Figure 6E*), making it difficult to determine whether NF^+^/TH^+^ fibres described originate from sympathomotor neurons of the SCG or the TH-expressing population of chemosensory neurons in the petrosal ganglion. However, based on observed fibre morphology and close association with CB vasculature, it is apparent that our analysis excluded adrenergic postganglionic terminals (*Figure 6B*), notwithstanding the expected neurofilament composition aforementioned earlier.^57^ This underscores the need for a comprehensive analysis regarding the origin of NF^+^/TH^+^ projections in the CB in future research.

A principal finding of the present study is the increased number of NF^+^ fibres in the CB of SHR (*Figure 5B* and *C*), indicating a net increase in CB innervation. This suggests increased arborization of the nerve fibres innervating the CB, i.e. an expanded per unit receptive field of arterial chemoafferents. Assuming that the number of chemosensory neurons in the petrosal ganglion and the number of sympathetic postganglionic neurons in the SCG innervating the CB are unchanged in the SHR. This highlights the action of neurotrophic factors promoting innervation of arterial chemoreceptors in hypertension. Published RNA-seq data indicate a substantial upregulation of brain-derived neurotrophic factor (*Bdnf*; FC: 1.92, P.adj = 0.0084) in the SHR CB.^36^ Similarly, Neurotrophin-3 (NT3, encoded by *Ntf3*), a known promoter of axonal arborization, exhibiting higher baseline expression in the CB compared with *Bdnf*, also showed an upward trend in expression in SHR (FC: 1.19, *P* = 0.039, P.adj = 0.143). This is further supported by an independent immunohistochemical study showing increased production of BDNF and NT3 in the SHR CBs.^22^ These data suggest that neurotrophic signalling promoting chemoafferent innervation may represent a potential therapeutic target for modulating CB activity in cardiometabolic disease associated with CB hypertrophy and peripheral chemoreflex sensitization.

Translational perspective Carotid body (CB) hypertrophy in hypertension is associated with chemosensory hyperplasia and angiogenesis, likely mediated by impaired HIF-PHD signalling in the SHR. We propose that CB size may serve as a candidate marker of chemoafferent sensitivity and the efficacy of therapies targeting the CB. However, further validation in humans is needed to support this link. Neurotrophic pathways promoting increased chemoafferent innervation in hypertension are proposed as a potential target for modulating CB activity in sympathetically mediated diseases.

## Supplementary Material

cvaf207_Supplementary_Data

## Data Availability

Deconvolved image data generated by the study are publicly available at Zenodo: [available upon acceptance]. Data used for statistical analysis and plotting are available as see Supplementary material online, *Dataset S1*. Code generated is available via github.com/audrysgp/pauza_et_al-2025.
