# Supplementary material for: Age-dependent remodelling of arterial chemoafferent innervation in hypertension
Source: Cardiovasc Res. 2025 Oct 29;121(15):2347–59. doi: 10.1093/cvr/cvaf207 (PMC12687876; doi:10.1093/cvr/cvaf207)
Supplement: cvaf207_Supplementary_Data [file cvaf207_supplementary_data.zip › manuscript_text_CVR-2025-0673R1_supplementary.docx]

**Age-dependent remodelling of arterial chemoafferent innervation in hypertension**

Short title: Carotid body morphometry SHR

Audrys G. Pauza, Igor Felippe, Iris de Laat, Olivia Gold, Xin Shen, Julian F.R. Paton

Manaaki Mānawa – The Centre for Heart Research, Department of Physiology, Faculty of Medical & Health Sciences, University of Auckland, Grafton, Auckland, New Zealand

Corresponding author:

Audrys G. Pauza, PhD

Manaaki Manawa- The Centre for Heart Research

Department of Physiology

Faculty of Medical and Health Sciences

University of Auckland

85 Park Road, Grafton, Auckland 1023, New Zealand

Telephone: +6421 022 70477

Email: audrys.pauza@auckland.ac.nz

SUPPLEMENTARY MATERIALS

Supplementary Figure legends

**Supplementary Figure 1**. *Nuclei quantification in TH^+^ (chemosensory) cell clusters*

The TH-immunoreactive area was defined using a threshold-based pixel classifier in QuPath providing a rough outline of the chemosensory clusters. DAPI-stained nuclei were classified using automated StarDist^27^ object segmentation. **A** – Low magnification of TH^+^ clusters. **B-C** – magnified view of the outlined region in A showing classifier results. **D** – Nuclei detected inside and outside TH^+^ clusters using StarDist.^27^ TH – tyrosine hydroxylase (green).

**Supplementary Figure 2**. *Microvascular network perfusing the carotid body*

Representative image from old Wistar and SHR animals showing outlined perimeter of the carotid body based on TH immunoreactivity. TH – tyrosine hydroxylase (green). TL/LEL – Tomato (*Lycopersicon esculentum*) lectin (white).

**Supplementary Figure 3**. *Intrinsic autonomic neurons in the carotid body*

Neuronal cell bodies were rarely observed at the periphery of the carotid body. They were distinguished by their large size and strong immunoreactivity for the 160 kDa neurofilament protein. Some NF^+^ autonomic neurons exhibited visible processes (arrowheads). TH – tyrosine hydroxylase (green). NF – 160 kDa neurofilament-M (magenta). TL/LEL – Tomato (*Lycopersicon esculentum*) lectin (white).

**Supplementary File 1**. *3D rendering of CB from a representative aged Wistar rat.*

**Supplementary File 2**. *3D rendering of CB from a representative aged SHR rat.*

**Supplementary Dataset 1.** Morphometric data of CB size and composition in SHR and Wistar rats.
